# Revealing the Shared Genetic Basis of Thermal Adaptation and Abdominal Adiposity in Wenchang Chicken Using Whole-Genome Structural Variation Analysis

**DOI:** 10.3390/ani16152417

**Published:** 2026-08-05

**Authors:** Yu Sun, Lihong Gu, Shihao Chen, Danfeng Cai, Zhen Zhou, Jiahao Li, Ziyu Sang, Shenghua Wei, Qinghua Nie, Dexiang Zhang

**Affiliations:** 1 State Key Laboratory of Swine and Poultry Breeding Industry, Guangdong Laboratory for Lingnan Modern Agriculture, Guangdong Provincial Key Lab of Agro-Animal Genomics and Molecular Breeding, Key Laboratory of Chicken Genetics, Breeding and Reproduction, Ministry of Agriculture and Rural Affairs, National-Local Joint Engineering Research Center for Livestock Breeding, College of Animal Science, South China Agricultural University, Guangzhou 510642, China; 20243139089@stu.scau.edu.cn (Y.S.); danfengcai@stu.scau.edu.cn (D.C.); zhouzhen@stu.scau.edu.cn (Z.Z.); jiahaoliscau@foxmail.com (J.L.); 20243139083@stu.scau.edu.cn (Z.S.); 18824848793@stu.scau.edu.cn (S.W.); nqinghua@scau.edu.cn (Q.N.); 2 Institute of Animal Science & Veterinary Medicine, Hainan Academy of Agricultural Sciences, Haikou 571100, China; nil2008@yeah.net; 3 College of Animal Science and Technology, Yangzhou University, Yangzhou 225009, China; mrrchen@yzu.edu.cn

**Keywords:** heat stress, abdominal fat, selection signature, pleiotropy, poultry, lipid metabolism

## Abstract

Environmental temperature significantly influences the evolutionary adaptation of poultry, while abdominal fat deposition represents a crucial economic trait affecting feed conversion efficiency and carcass quality. However, reports on the genetic mechanisms governing their co-evolutionary trade-offs remain scarce. This study employed whole-genome resequencing data from 469 chickens across 31 varieties. Through comparative analyses of heat- versus cold-adapted groups and high- versus low-fat groups, structural variations (SVs) were utilized as genetic markers. Selection signatures were identified via the population differentiation index (*F_ST_*) and nucleotide diversity ratio (π ratio). We identified 103 overlapping genes located within significantly differentiated SVs at the intersection of temperature adaptation and fat deposition. Pathway analysis revealed significant enrichment in the thyroid hormone signaling pathway, pinpointing *MED17* as a key selection target. Further validation via PCR genotyping in the heat-tolerant Wenchang chicken revealed that individuals harboring the *MED17* mutation exhibited significantly higher abdominal fat deposition than those without. These results suggest that *MED17* is associated with fat deposition in heat-tolerant Wenchang chickens, serving as a potential candidate gene for this trait, providing robust molecular markers for breeding novel strains with both thermotolerance and superior carcass traits.

## Simple Summary

Global warming challenges poultry farming by inducing heat stress, which alters how chickens store fat and reduces meat quality. To resolve the conflict between thermal adaptation and fat deposition, we analyzed large-scale DNA changes, known as genomic structural variations, across diverse chicken breeds. We discovered that specific genetic changes, particularly in the *MED17* gene, show a candidate association with both traits. These findings provide poultry breeders and the agricultural sector with precise genetic markers. Utilizing these tools allows for the breeding of novel, heat-resilient chickens that maintain high-quality meat. This ultimately benefits farmers economically and ensures sustainable poultry production in increasingly hot climates.

## 1. Introduction

While global livestock production has increasingly shifted toward developing countries in tropical and subtropical regions, the full economic potential of this expansion is severely constrained by high-temperature environments. Consequently, heat stress adversely impacts livestock production performance [1], causing substantial economic losses [2]. As homeotherms, poultry adjust their energy allocation during heat stress, redirecting metabolic energy toward thermoregulation. Prolonged heat stress compromises the intestinal barrier, immune function, and metabolism of poultry, inducing oxidative stress and metabolic disorders, ultimately impeding protein deposition and promoting abnormal fat accumulation [3]. Abdominal fat percentage is a critical economic trait for assessing the carcass quality of broilers and exhibits a significant negative correlation with feed conversion ratio.

Heat stress orchestrates endocrine shifts by activating the hypothalamic–pituitary–adrenal (HPA) axis, which promotes lipid redistribution and excessive abdominal fat deposition, thereby exacerbating physiological dysfunction in broilers [4,5,6,7].

Broiler abdominal fat deposition is dynamically regulated by complex hepato-adipose metabolic networks (e.g., PPARs and CPT) [8]. Multi-omics evidence reveals that heat stress drives neuroendocrine–lipid crosstalk, specifically altering the PPAR pathway, to disrupt molecular lipid homeostasis [9].

However, the elusive genetic basis of abdominal fat deposition associated with climate adaptation hinders marker-assisted breeding in poultry [10]. Therefore, deciphering the genetic link between thermotolerance and adiposity is crucial for developing stress-resilient, efficient breeds.

Modern commercial broiler chicken strains, characterized by their exceptionally high metabolic rates, are highly susceptible to respiratory alkalosis and endocrine axis disorders, with mortality rates reaching up to 20% under extreme heat conditions [11]. This underscores the urgent need to uncover natural heat-tolerant genetic resources within indigenous breeds. Traditional poultry genetics research primarily utilizes SNPs to investigate trait association loci. In contrast, SVs, which represent a prevalent form of large-scale genomic variation, offer broader genomic coverage and exert more significant phenotypic effects than SNPs [12,13]. Recent research suggests that constructing comprehensive population-level SV maps is essential for a thorough analysis of genetic diversity among species and for identifying genes linked to complex phenotypes [14]. Furthermore, SVs capture crucial genetic variation that traditional SNP arrays often miss. Integrating SVs into genomic analyses can substantially enhance the resolution of complex trait mapping and improve the accuracy of genomic selection [15]. Recent pan-genome studies demonstrate that SVs can directly alter gene dosage or modify cis-regulatory elements. These structural changes play an essential role in shaping complex traits and driving adaptive evolution to extreme environments in livestock [16]. In poultry, the advent of telomere-to-telomere (T2T) pangenomes has highlighted the critical role of structural variations (SVs) in deciphering complex traits by uncovering hidden variants in previously unresolved regions [17]. Rather than merely serving as neutral markers, specific SVs have been demonstrated to directly drive phenotypic divergence. Recent pan-genomic studies have successfully identified large-scale variants as causal mutations for key economic traits, including carcass yield, muscle development, and meat quality myopathies [17,18,19]. Furthermore, complex nested SVs play pivotal roles in breed-specific adaptations and morphological traits [19]. Incorporating these SV-level insights into genetic analyses not only captures a substantial proportion of missing heritability (uncovering nearly 30% of novel QTLs [17]), but also significantly enhances the predictive accuracy of genomic selection models compared to traditional SNP arrays [18]. Therefore, performing selection signature analysis using genome-wide SVs will elucidate the intricate genetic architecture underlying thermotolerance and abdominal fat deposition in poultry.

This study integrated high-depth (30×) whole-genome resequencing data from 31 chicken breeds, encompassing commercial lines, indigenous breeds, and wild ancestors. Variant calling yielded a robust dataset of high-quality SVs. Using these SVs, we analyzed population structure and selection signatures, identifying 317 genes associated with abdominal fat deposition and 270 genes linked to temperature adaptation. Functional enrichment analysis (GO/KEGG) systematically highlighted the specific biological pathways regulating these complex traits. Notably, the intersection of these datasets revealed 103 overlapping genes. Hypergeometric testing demonstrated that this overlap between the two gene sets was significantly greater than expected by chance (*p* = 2.87 × 10^−118^), suggesting a shared genetic basis for adiposity and environmental adaptation. To validate these findings, we performed PCR genotyping and phenotypic association analyses on targeted SV sites within candidate genes, such as *MED17*, in a validation population. Ultimately, this research not only provides a novel structural genomic perspective on the shared adaptive mechanisms underlying complex traits in poultry, but also offers precise genetic markers to breed novel strains with robust thermotolerance and superior meat quality.

## 2. Materials and Methods

### 2.1. Ethics Statement

All animal experiments performed in this study satisfied the requirements of the Institutional Animal Care and Use Committee at South China Agricultural University (Approval ID: SCAU-2024F018), with due consideration given to the 3Rs (Replacement, Reduction, and Refinement). Experimental animals were slaughtered following the Chinese national standard (GB/T19478-2018) [20]. The reporting of this study is in accordance with the ARRIVE guidelines.

### 2.2. Study Design and Sample Collection

The WGS data analyzed here include newly generated in-house datasets and public data from NCBI. This comprehensive dataset comprises paired-end sequencing data from 469 individuals across 31 populations—including indigenous breeds, commercial strains, and wild ancestors—with an average sequencing depth of approximately 30×. Detailed breed information and sample sizes are provided in Table 1; the specific accession numbers and data references for all sequenced individuals are listed in Table S1.

### 2.3. Quality Control, Sequence Alignment, and Structural Variant Calling

To ensure accurate structural variant (SV) detection, stringent quality control was applied to the raw reads. First, fastp (v0.19.4) [21] was used to remove adapter sequences, low-quality reads, and reads with excessive unknown bases (N). The resulting clean reads were aligned to the chicken reference genome (GRCg7b) using Sentieon (v201711.02) [22]. PCR duplicates were then marked and removed to generate high-quality BAM files. Structural variants were called from these deduplicated alignment files using Delly (v1.0.3) [23]. Following SV calling, we applied rigorous filtering to ensure a high-confidence dataset. Specifically, we retained SVs that met the following criteria: (1) Genotype Quality (GQ) > 20; (2) Read Depth (DP) ≥ 10; (3) variant call rate > 90% (missing rate < 10%); (4) SV length ≥ 50 bp; and (5) “PASS” filter status in the VCF file. These thresholds were chosen to ensure data reliability and reproducibility. The resulting high-confidence SV dataset was then used for downstream population genetics and selection signature analyses.

### 2.4. Population Genetics Analysis

To assess population structure and construct a phylogenetic tree, a total of 13,185 high-confidence SVs from the 31 populations were retained after filtering using PLINK v1.9.0 (--geno 0.2 --maf 0.05). A neighbor-joining (NJ) tree was constructed using VCF2Dis and visualized with iTOL. Principal component analysis (PCA) was performed in PLINK, and population admixture was inferred using the ADMIXTURE program (v1.3.0).

### 2.5. Population Grouping and Genome-Wide Selection Signature Analysis

For the abdominal fat deposition trait, we utilized historical slaughter performance data retrieved from Animal Genetic Resources in China: Poultry. To establish a robust phenotypic contrast, data for highly commercialized breeds, renowned for their extremely low abdominal fat content, were incorporated from the published literature and public repositories. To minimize potential biases from historical recording methods, representative breeds exhibiting extreme phenotypic divergence were selected to establish high-fat and low-fat groups using a 4% abdominal fat percentage (AFP) threshold (Table 2). This extreme phenotype design maximizes inter-group genetic differentiation, thereby improving the detection power for SVs associated with lipid metabolism.

To identify selection signatures associated with thermal adaptation, the 31 populations were geographically divided by the Qinling–Huaihe River line, a major climatic boundary in China. The region south of this line exhibits a subtropical monsoon climate (high temperature and humidity), whereas the north experiences a temperate continental monsoon climate (cold, dry winters). Indigenous chicken breeds evolving on either side of this boundary have faced distinct environmental selection pressures. This macro-ecological dichotomy provides a robust binary framework for detecting historical signatures of thermal adaptation (Table 2)

Fixation index (*F_ST_*) and nucleotide diversity (π) values for the SV loci were calculated using VCFtools (v0.1.17). We subsequently identified selection signatures by intersecting genomic regions that exhibited extreme *F_ST_* values and highly divergent nucleotide diversity ratios log_2_(π_group1_/π_group2_) allowed us to reliably pinpoint significantly selected SV loci.

### 2.6. Gene Function Annotation and Pathway Enrichment Analysis

For functional annotation, the genomic coordinates of significant structural variant (SV) loci were mapped against the chicken reference genome (GRCg7b) using a nearest gene annotation approach. Briefly, the absolute physical distances between each SV coordinate and the boundaries of all annotated genes within the same chromosome were calculated using custom R scripts (utilizing the dplyr package, v1.2.1), and the gene with the minimum distance was assigned as the target candidate gene for the respective SV. Subsequently, Gene Ontology (GO) and Kyoto Encyclopedia of Genes and Genomes (KEGG) pathway enrichment analyses were conducted using the ShinyGO 0.85 web server based on these identified candidate genes. Statistical significance for enriched pathways was defined as a false discovery rate (FDR) < 0.05.

### 2.7. Validation of SVs

To experimentally validate the candidate SVs and determine their genotypes within the population, genomic DNA was extracted from Wenchang chicken (WCC) blood samples using a commercial nucleic acid extraction kit (Cat. #051607-2A96, Cyagen Biosciences, Guangzhou, China). PCR primers were specifically designed to flank the breakpoints of the candidate SVs, targeting the *MED17* gene. The primer sequences used to genotype the 166 bp deletion were: forward, 5′-TAT CCT CAT TCC GTG AAA GCA GT-3′; and reverse, 5′-AAG AGG AAT CCG TGG GTA TTC AG-3′. PCR amplification was performed in a 30 µL reaction volume containing 1.8 µL template DNA, 25.8 µL 1.1× S4 Fidelity PCR Mix (Cat. #SF212, Fidelity Enzyme, Guangzhou, China), and 1.2 µL of each primer. Thermocycling conditions included an initial denaturation at 98 °C for 2 min; 34 cycles of denaturation at 98 °C for 10 s; annealing at 61 °C for 15 s; extension at 72 °C for 15 s; followed by a final extension at 72 °C for 5 min. PCR products were stored at 4 °C. Subsequently, the PCR products were resolved on agarose gels (Cat. #TSJ001, Tsingke Biotechnology, Beijing, China) alongside a standard DNA marker (Cat. #BM101-01, TransGen Biotech, Beijing, China) to verify amplification specificity. Finally, the validated PCR products were sent to Guangzhou Tianyi Huiyuan Gene Technology Co., Ltd. (Guangzhou, China) for Sanger sequencing to precisely identify the SV genotypes and breakpoints.

### 2.8. Statistical Analysis

All statistical analyses and graphical visualizations were conducted using R software (v4.4.2), and IBM SPSS Statistics (version 27.0; IBM Corp., Armonk, NY, USA). For the genome-wide selection signature analysis, genomic regions within the top 5% of the empirical distributions for both *F_ST_* and log_2_(π ratio) were defined as significant candidate regions. Based on these selection signatures, *MED17* was identified as a key candidate gene. Subsequently, to evaluate phenotypic differences in abdominal fat traits, the association between the *MED17* genotypes and these traits was analyzed using a General Linear Model (GLM) in SPSS. Because all experimental animals were female Wenchang chickens raised in the same batch and slaughtered at the same age, the effects of sex, age, and batch were uniform. Body weight was included as a continuous covariate to adjust for underlying physiological differences. The mathematical model was as follows:

(1) $$\begin{array}{c} Y_{ij}=\;\mu +\;G_{i}+\;bW_{ij}+\;e_{ij},\; \end{array}$$

where $Y_{ij}$ is the observed phenotypic value of the trait; *µ* is the overall population mean; $G_{i}$ is the fixed effect of the *MED17* genotype; b is the regression coefficient for body weight; $W_{ij}$ is the observed body weight used as a covariate; and $e_{ij}$ is the random residual error. Data are presented as mean ± standard error of the mean (SEM), and statistical significance was set at *p* < 0.05.

## 3. Results

### 3.1. Genome-Wide SV Characterization and Population Genetic Structure of Diverse Chicken Breeds

#### 3.1.1. Population Genetic Structure and Shared Ancestry

**Figure 1:**
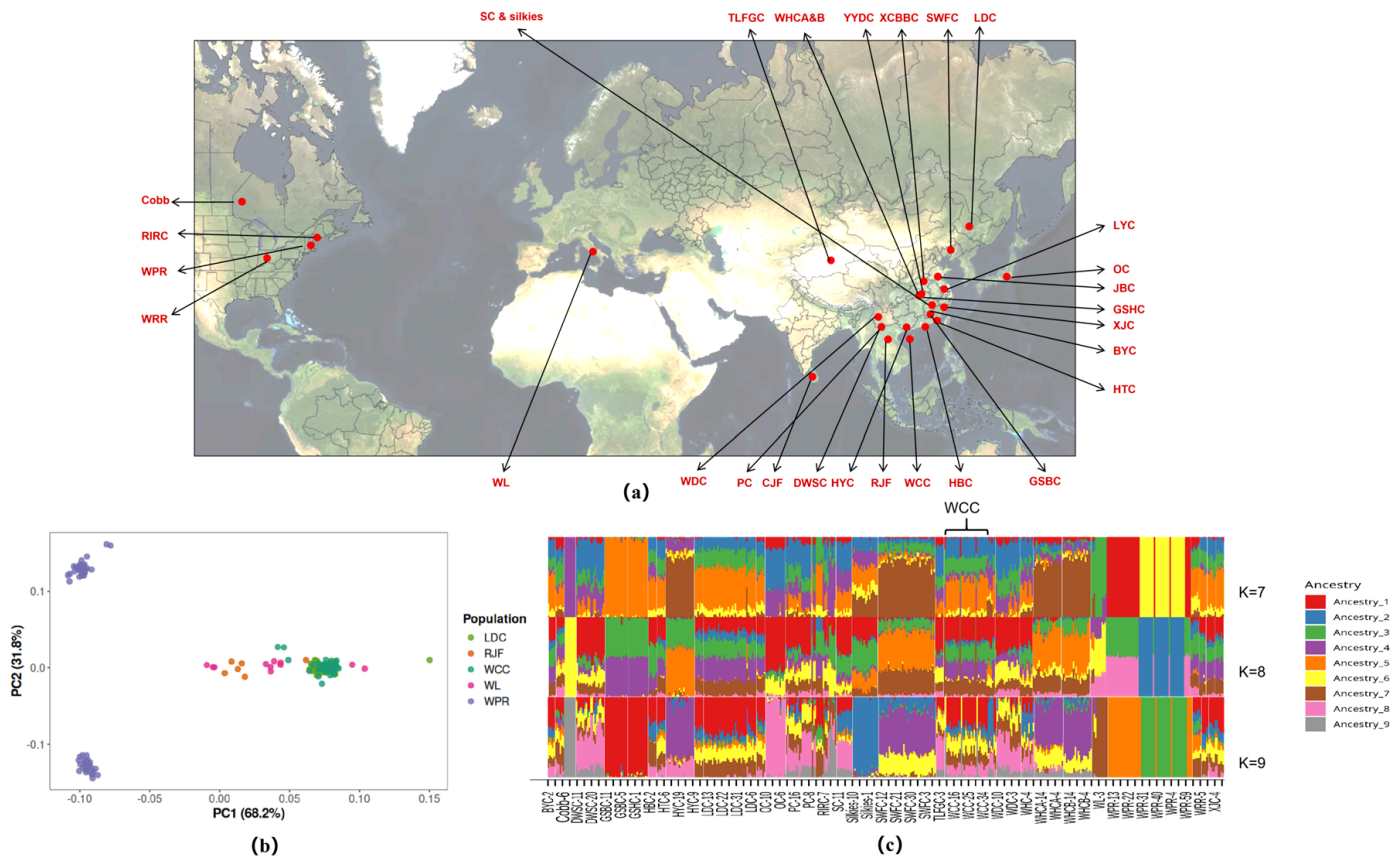
Geographic distribution and population genetic structure of the studied chicken breeds. (**a**) Geographic distribution of the 31 sampled chicken populations worldwide. (**b**) Focused Principal Component Analysis (PCA) illustrating the genetic stratification of the five focal populations. (**c**) Admixture analysis showing the population structure and genetic proportions at different K values (K = 7 to 9).

To elucidate the genetic and evolutionary relationships among chicken breeds adapted to diverse thermal environments, we analyzed whole-genome resequencing data from 469 individuals across 31 global populations (Figure 1a). An initial principal component analysis (PCA) of all sampled populations revealed distinct genetic structures (Figure S1). The first two principal components (PC1 and PC2) accounted for 43.9% and 22.2% of the genetic variance, respectively (cumulative variance: 66.1%). A focused PCA (Figure 1b) revealed distinct genetic stratification: WPR formed an isolated cluster at the negative extreme of the PC1 axis, whereas RJF and WL occupied intermediate positions. Notably, WCC and LDC clustered tightly at the far positive end, reflecting their shared genomic background as indigenous Chinese breeds. This high genomic similarity suggests that their evident phenotypic divergence is likely driven by localized selection pressures rather than broad ancestral differences. Furthermore, Admixture analysis provided a quantitative assessment of population structure and shared ancestry (Figure 1c). Based on cross-validation (CV) error calculations [24], the optimal number of ancestral lineages was determined to be *K* = 8 (Figure S1). Under this optimal model, the population structure closely aligned with the findings from the PCA and phylogenetic clustering. Highly commercialized breeds (e.g., WPR) displayed a highly homogeneous, single ancestral component. In contrast, the majority of indigenous breeds, including WCC, exhibited a diverse array of genetic compositions. At *K* = 8, genomic profiling confirmed that the WCC population harbors components from multiple ancestral sources, thereby maintaining a remarkably high level of genetic diversity.

#### 3.1.2. Genomic SV Profiling and Phenotypic Characterization

**Figure 2:**
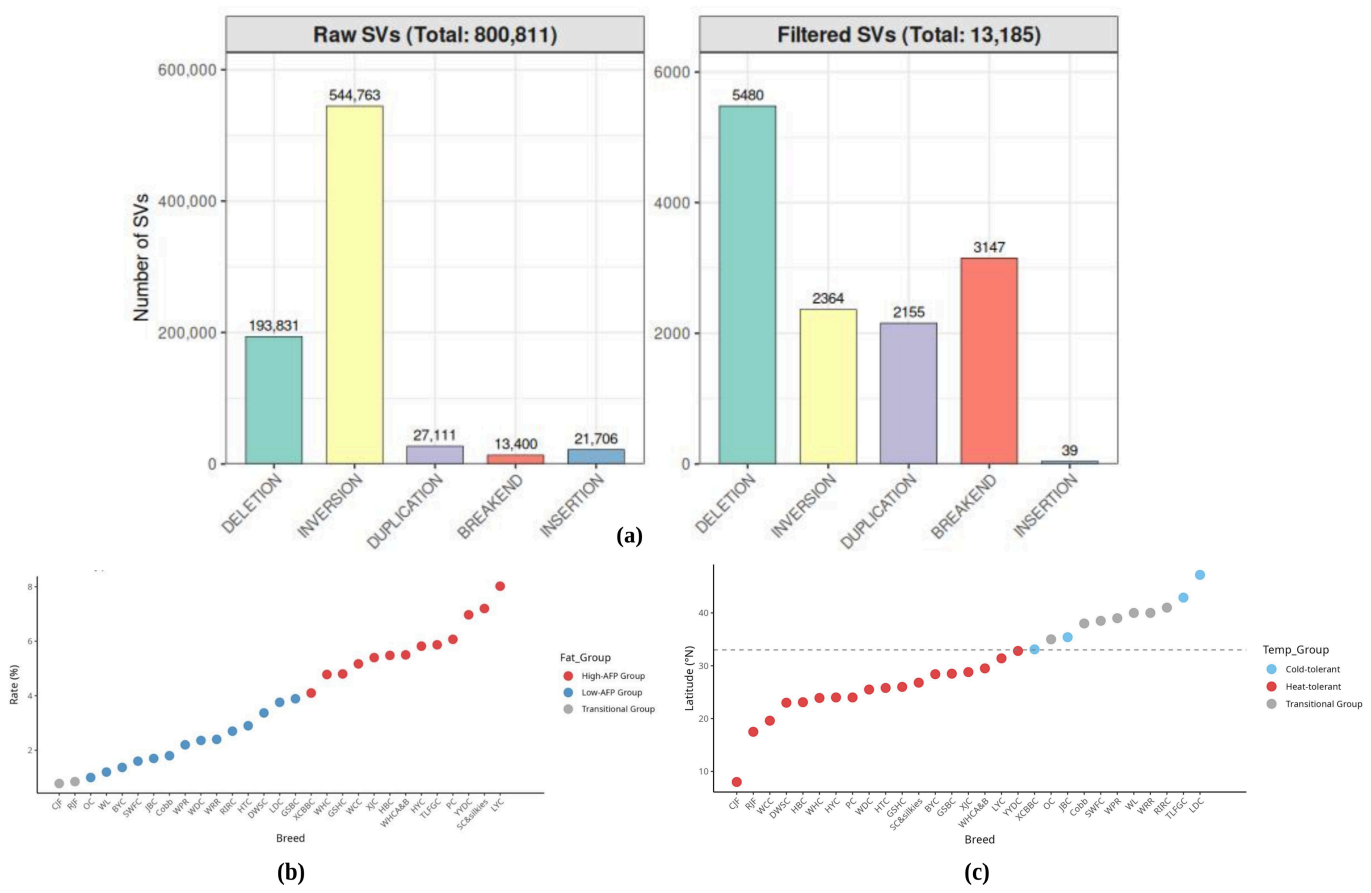
Genomic structural variations and functional adaptation characteristics across different chicken breeds. (**a**) Summary of the quantity and types of structural variations (SVs), displaying both the raw dataset (**left**) and the strictly filtered SVs (**right**). (**b**) Phenotypic distribution of populations ordered by their mean abdominal fat percentage (AFP). Breeds are color-coded based on their phenotypic classification: High-AFP (red), Low-AFP (blue), and Transitional (grey) groups, with wild ancestors also included. (**c**) Latitudinal distribution of populations ordered by their origin (decimal degrees North). The dashed line indicates the Qinling–Huaihe Line (~33° N), the traditional geographic boundary between North and South China. Colors represent environmental adaptation groups: Heat-tolerant (red), Cold-tolerant (light blue), and Transitional (grey).

To investigate the genomic structural variations (SVs), we first conducted a comprehensive SV profiling. A total of 800,811 raw SVs were initially identified, which were predominantly composed of inversions (544,763) and deletions (193,831). Following stringent quality control and filtering criteria, a robust dataset of 13,185 high-quality SVs was retained for downstream analysis. Within this filtered dataset, deletions remained the most abundant SV type (5480), followed by breakends (3147), inversions (2364), and duplications (2155) (Figure 2a Table S2). Furthermore, we characterized the distinct phenotypic and environmental traits of these breeds. The assessment of abdominal fat percentage (AFP) revealed a continuous phenotypic distribution across the 31 breeds, allowing for their categorization into a low-AFP group, a transitional group, and a high-AFP group (Figure 2b). Concurrently, an analysis of their latitudinal distribution demonstrated a clear geographical divergence associated with environmental adaptation. Breeds predominantly located at lower latitudes were classified as heat-tolerant, whereas those distributed at higher latitudes (above approximately 33° N) exhibited cold-tolerant characteristics (Figure 2c).

#### 3.1.3. Phylogenetic Evolution Patterns Driven by Thermal Adaptation

**Figure 3:**
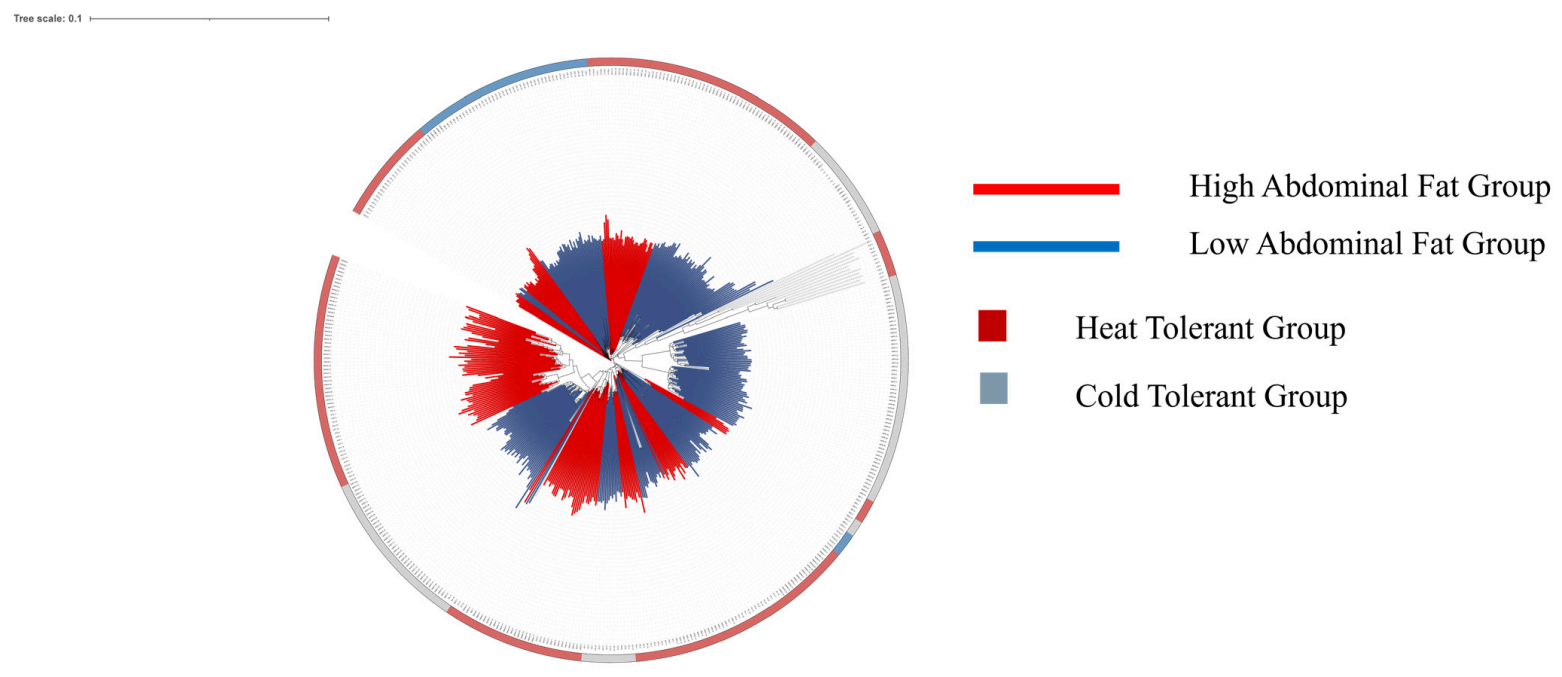
Phylogenetic evolution patterns of chicken breeds based on structural variations. Circular neighbor-joining (NJ) tree constructed from whole-genome structural variations (SVs). The tree is annotated with phenotypic and environmental traits: abdominal fat percentage (outer layer; red lines: high-fat, blue lines: low-fat) and temperature adaptation (inner squares; red: heat-tolerant, blue/grey: cold-tolerant). The scale bar represents genetic distance (0.1).

Based on the whole-genome SVs, we constructed a neighbor-joining (NJ) tree annotated with phenotypic traits (Figure 3). The results revealed that lineages characterized by high (red) and low (blue) abdominal fat did not form distinct clusters but rather exhibited a mixed distribution. This topological admixture is consistent with the highly polygenic nature of abdominal fat deposition. Furthermore, because an SV-based phylogeny primarily reflects demographic history and genome-wide divergence, the sporadic distribution of abdominal fat phenotypes across diverse lineages likely results from independent parallel evolution under specific selection pressures. In contrast, clustering by temperature adaptability (heat-tolerant in red, cold-tolerant in blue, transitional in gray) displayed pronounced regional aggregation with relatively continuous clades. This observation indicates that phylogenetic clustering patterns in these breeds are more heavily driven by thermal adaptation than by fat deposition.

### 3.2. Candidate Gene Identification and Functional Enrichment Based on Selection Signatures

**Figure 4:**
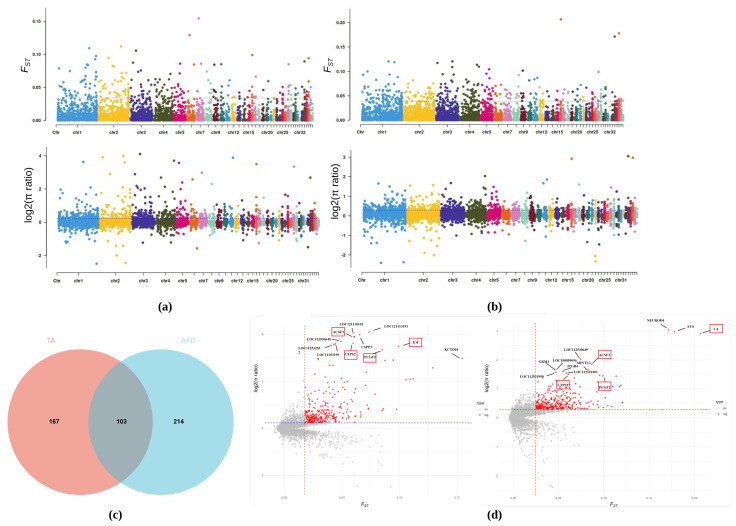
Genomic signatures of selection associated with thermal adaptation and abdominal fat deposition. (**a**) Manhattan plots of *F_ST_* (**top**) and log_2_(π ratio) (**bottom**) across the genome between the heat- and cold-tolerant groups. (**b**) Corresponding Manhattan plots identifying selective sweeps between the high- and low-abdominal fat groups. (**c**) Venn diagram detailing the overlap of candidate genes under positive selection in both the thermal adaptation (TA) and abdominal fat deposition (AFD) analyses. (**d**) 2D scatter plots of log_2_(π ratio) versus *F_ST_* (top). The red boxes specifically highlight the shared core candidate genes that are co-selected in both independent analyses. Note: In the Manhattan plots (**a**,**b**), each data point represents an individual SV region. In contrast, the numbers in the Venn diagram (**c**) and the plotted points in the scatter plots (**d**) represent annotated candidate genes.

**Figure 5:**
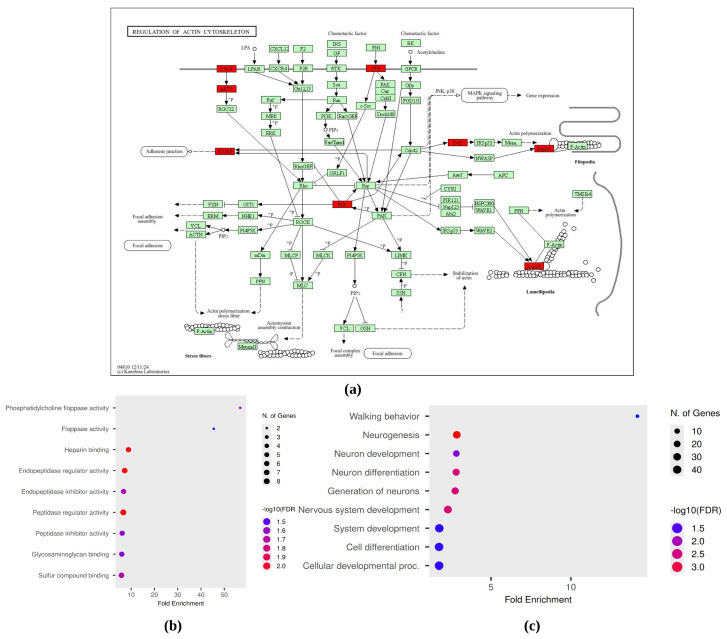
Functional enrichment and pathway analysis of the candidate genes. (**a**) KEGG pathway diagram illustrating the involvement of candidate genes in the regulation of the actin cytoskeleton. Red nodes highlight targets identified in the temperature-adapted group. (**b**) Gene Ontology (GO) molecular function enrichment analysis of candidate genes associated with thermal adaptation, notably highlighting floppase and peptidase activities. (**c**) GO biological process enrichment analysis demonstrating pathways associated with divergent abdominal fat deposition, particularly walking behavior and neurogenesis.

In this study, we identified 270 candidate genes associated with temperature adaptation and 317 genes associated with abdominal fat deposition based on *F_ST_* and π ratio selection signatures (Figure 4a,b; Table S3). Intersection analysis of these two datasets revealed 103 overlapping genes (Figure 4c; Table S4). Among the top 10 selection signatures identified for both traits, four genes *ACSF3*, *CTPS2*, *PCGF2*, and *C4*) overlapped, suggesting they are primary targets of selection (Figure 4d). Based on their established biological roles, these genes represent functional candidates linking lipid homeostasis (e.g., *ACSF3* in fatty acid synthesis) and cellular stress responses (e.g., *PCGF2* and *CTPS2*) to environmental adaptation. KEGG pathway analysis indicated that candidate genes related to temperature adaptation were significantly enriched in the regulation of actin cytoskeleton pathway (Figure S3A), which includes nine key genes such as *ITG*, *AKT1*, and *PIX* (Figure 5a). GO functional annotation demonstrated that these selected genes were significantly enriched in cell surface components (Figure S3B). Regarding molecular functions, significant enrichment was primarily observed in enzyme activity regulation, lipid transmembrane transport, and macromolecular binding (Figure 5b). Furthermore, candidate genes exhibiting strong selection signals between the high and low abdominal fat groups showed significant enrichment in walking behavior—a key component of overall locomotor activity—as well as several neural-related developmental processes such as neurogenesis and neuron differentiation (Figure 5c), including four key genes: *LARGE1*, *KLHL1*, *UCHL1*, and *DAB1*. These significantly enriched GO terms and pathways (*p* < 0.05) are detailed in Table S5.

### 3.3. Association Analysis Between the MED17 SV Locus and Abdominal Fat Traits

**Figure 6:**
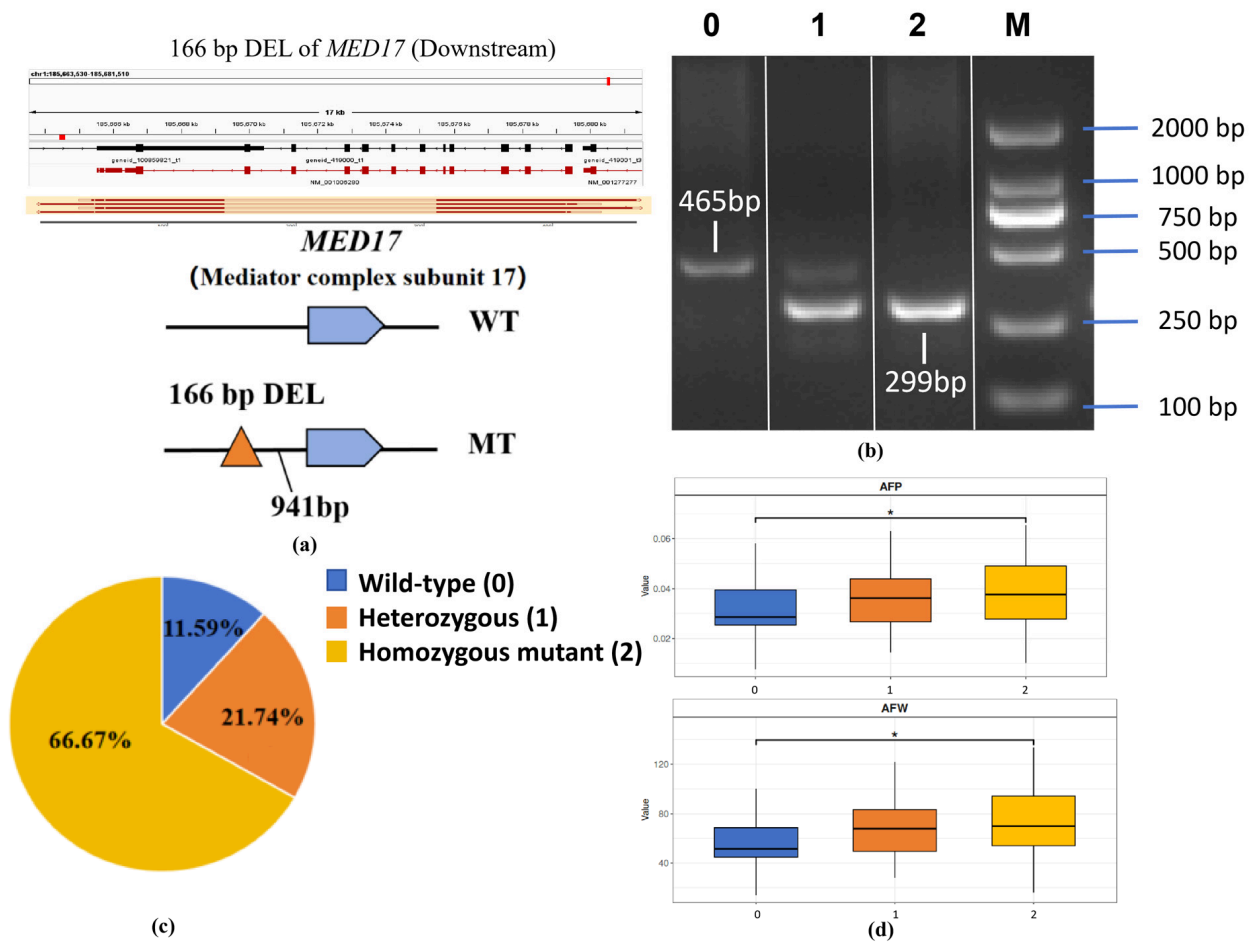
Identification and phenotypic association of the 166 bp deletion in *MED17*. (**a**) Genomic mapping and schematic diagram of the 166 bp deletion (DEL) structural variation within the *MED17* locus, illustrating the wild-type (WT) and mutant (MT) alleles. (**b**) PCR amplification and gel electrophoresis confirming the three genotypes: homozygous wild-type (0), heterozygous (1), and homozygous mutant (2). Vertical lines indicate where lanes from the same gel have been rearranged for clarity. (**c**) Genotype frequency distribution within the natural validation population of 207 WCC individuals. (**d**) Boxplots demonstrating the significant associations of the *MED17* genotypes with abdominal fat percentage (AFP) and abdominal fat weight (AFW). Asterisk (*) indicate significant differences between genotypes (*p* < 0.05).

Among the 103 overlapping genes, *MED17* was selected as the primary target for functional validation due to its core pleiotropic roles in regulating lipid metabolism, endocrine signaling, and environmental stress responses. We focused on a specific 166 bp deletion (SV) located downstream of the *MED17* locus (Figure 6a). To validate its genetic effects, PCR genotyping was conducted across a population of 207 WCC individuals. Gel electrophoresis successfully distinguished three distinct genotypes: wild-type (0), heterozygous (1), and homozygous mutant (2) (Figure 6b). Representative original gel electrophoresis images are provided in Figure S4. Genotype frequency analysis revealed that the homozygous mutant genotype (2) was the predominant genotype, accounting for 66.67% of the population, followed by the heterozygous (1) and wild-type (0) genotypes (Figure 6c). Crucially, phenotypic association analysis demonstrated that abdominal fat traits were significantly associated with the *MED17* downstream 166 bp DEL variant genotypes (Table 3). Individuals carrying the homozygous mutant genotype (2) exhibited significantly higher abdominal fat percentage (AFP) and abdominal fat weight (AFW) compared to the wild-type (0) and heterozygous (1) genotypes (Table 3; *p* < 0.05; different lowercase letters indicate significant differences; Figure 6d).

## 4. Discussion

Utilizing genome-wide SVs, this study highlights the distinct genomic impacts of natural and artificial selection on poultry. Unlike commercial breeds [25,26], the WCC population maintains robust polymorphism and a deeply admixed architecture [27,28]. WCC preserves substantial variation in abdominal fat and heat tolerance, serving as an ideal model for decoding complex economic traits. Phylogenetic analysis shows that abdominal fat phenotypes evolved independently [29]. Conversely, temperature-adaptive traits exhibit strong geographical clustering, driven by convergent evolution in similar ecological zones [30].

Cross-trait analysis revealed a significant overlap of 103 candidate genes. From an evolutionary perspective, this reflects a crucial energy allocation strategy in high-temperature environments. Specifically, mitigating heat stress necessitates a reduction in endogenous heat production by depressing basal metabolism; this physiological adjustment often requires reallocating conserved energy toward lipid biosynthesis. Previous studies support this paradigm: extreme heat compels poultry to reconfigure metabolic pathways, concurrently enhancing de novo lipogenesis [31].

We identified four core genes—*ACSF3*, *CTPS2*, *PCGF2*, and *C4*—as pivotal molecular hubs. *ACSF3* encodes a crucial enzyme in mitochondrial de novo fatty acid synthesis, providing essential lipid precursors [32,33]. Concurrently, selective pressure on *CTPS2* likely reflects a mechanism to curtail energy-intensive cell proliferation, thereby reducing overall thermogenesis [34]. Despite being biochemically independent, these 103 pleiotropic genes serve as a molecular bridge linking environmental thermal adaptation to systemic shifts in lipid metabolism.

Pathway analysis showed that temperature-adaptation-associated genes (e.g., *ITG* family, *AKT1*, *Arp2/3*) are enriched in actin cytoskeleton regulation. This suggests an adaptive response to heat-induced cellular damage [35,36], where membrane-localized integrins detect external stress to trigger anti-apoptotic effects and actin repolymerization [37,38].

Furthermore, genes differentiating abdominal fat groups were enriched in CNS pathways governing walking behavior (e.g., *LARGE1*, *DAB1*). This implies that populations with heightened locomotor activity expend more energy, thereby restricting the surplus available for the abdominal fat pool [39,40,41,42,43,44,45].

Cross-population analysis identified *MED17* as a core candidate for both traits. PCR genotyping and subsequent association analysis revealed a significant correlation between the *MED17* variant and abdominal fat deposition in WCC. Premium meat quality relies on extensive lipid deposition. Because abdominal fat positively correlates with palatability-determining intramuscular fat, traditional selection for flavor likely drove the inadvertent co-selection and rapid enrichment of the *MED17* mutation. Consequently, high abdominal fat represents a physiological byproduct of this selection. Furthermore, the pleiotropic role of *MED17* in both thermal adaptation and fat metabolism likely provides a survival advantage under chronic heat stress. In vitro experiments demonstrate that *MED17* modulates adipocyte differentiation by interacting with transcription factors to upregulate key lipogenic genes, notably *FASN* [46]. We hypothesize that the *MED17* SV rewires interaction networks—specifically distal enhancer–promoter loops—to amplify downstream targets like *FASN*, driving excessive abdominal fat deposition.

Beyond lipid biosynthesis, *MED17* exhibits striking pleiotropy in regulating CNS-mediated motor behavior and fat deposition. This is supported by its robust transcriptional profile across tissues (Figure S5), which shows substantial expression of *MED17* in central nervous tissues and adipose tissue. Under chronic heat stress, reducing spontaneous locomotion and suppressing the hypothalamic–pituitary–thyroid (HPT) axis are vital strategies to minimize basal thermogenesis. Consistent with the strong positive selection of *TSHR* observed in WCC, *MED17* acts as a crucial coactivator within the thyroid hormone receptor (TR) network to suppress the HPT axis [47,48]

Mechanistically, network analysis integrates *MED17* into a temperature-driven “membrane–cytoskeleton–nuclear” axis (Figure S4). Under extreme heat, the membrane-localized ITGA/B complex transduces stress signals via cytoskeletal (e.g., Arp2/3) and kinase cascades that converge on the *MED17* nuclear hub. Responding to these environmental and endocrine cues, the *MED17* variant alters the Mediator complex’s regulatory activity to potently activate lipogenic genes like *FASN* [49].

Drawing on 3D genomics, we further hypothesize that the *MED17* SV functions as a distal enhancer [50]. This variant likely disrupts preexisting enhancer–promoter networks, creating topological alterations that upregulate downstream targets (e.g., *ABCB1*) to drive excessive triglyceride accumulation.

Despite these insights, several technical and experimental limitations should be noted. Stringent filtering substantially reduced initial SVs, reflecting strong purifying selection against these deleterious variants [19,51], while the commercial reference (GRCg7b) inherently biased variant calling toward deletions over insertions [52]. Furthermore, short-read sequencing is inherently constrained in accurately resolving complex variants [18]. Experimentally, the relatively small sample sizes for certain indigenous breeds might limit the detection of ultra-rare alleles, and the binary Qinling–Huaihe ecological boundary oversimplifies continuous climatic gradients. Additionally, compiling historical phenotypic records inherently introduces potential confounding from variations in management practices, and the precise cellular mechanism of the *MED17* pathway warrants deeper functional validation. Future research incorporating long-read pangenomics, landscape genomics with continuous bioclimatic variables, synchronized feeding trials, and downstream in vitro assays will be essential to rigorously expand and validate these preliminary markers.

## 5. Conclusions

Based on high-depth (30×) resequencing data from 469 chickens across 31 breeds, this study characterized genome-wide structural variations (SVs) to elucidate the genetic links between thermal adaptation and abdominal fat deposition. Selection signature analysis revealed 103 overlapping genes that are significantly enriched in the thyroid hormone signaling pathway. Notably, *MED17* emerged as a key candidate gene mediating the crosstalk between lipid metabolism and heat stress; its downstream 166 bp deletion was experimentally validated via PCR to be significantly associated with abdominal fat traits (*p* < 0.05). Ultimately, these SV markers provide an important theoretical foundation for the genetic breeding of stress-resistant traits in heat-tolerant poultry breeds, represented by Wenchang chickens.

## Figures and Tables

**Table 1:** Detailed sample information of the 31 chicken populations analyzed in this study.

Abbreviation	Breed Name	Sample Size
BYC	Baier Yellow chicken	5
Cobb	Cobb Broiler	6
CJF	Ceylon Junglefowl	8
DWSC	Daweishan Mini Chicken	20
GSBC	Green-shell laying hens with black feather	16
GSHC	Green-shell laying hens with hemp feather	14
HBC	Huiyang Beard Chicken	6
HTC	Hetian Chicken	6
HYC	Huangyao Chicken	20
JBC	Jining Bairi Chicken	6
LDC	Lindian Chicken	37
LYC	Liyang Chicken	6
OC	Oshamo	14
PC	Piao Chicken	18
RIRC	Rhode Island Red	11
RJF	Red Junglefowl	6
SC	Silkies	11
Silkies	Silkies	20
SWFC	Small White-feathered Chicken	40
TLFGC	Tulufan Game Chicken	6
WCC	Wenchang Chicken	36
WDC	Wuding Chicken	17
WHC	Wuhua Chicken	9
WHCA	Wuhei Chicken Line A	20
WHCB	Wuhei Chicken Line B	20
WL	White Leghorn	11
WPR	White Plymouth Rock	59
WRR	White Recessive Rock	6
XCBBC	Xichuan Black-bone Chicken	5
XJC	Xianju Chicken	6
YYDC	Yunyang Da Chicken	6

Detailed sample information of the 31 chicken populations analyzed in this study. This file provides a comprehensive overview of the samples used in the genomic analysis, including the abbreviation, full breed name, and sample size for each population. Notably, ‘SC’ (retrieved from NCBI) and ‘Silkies’ (newly sequenced in-house) represent two distinct datasets of the Silkie breed, while ‘WHCA’ and ‘WHCB’ designate two specialized breeding lines (Line A and Line B) of the Wuhei chicken. To ensure data origin transparency and preserve line-specific genomic characteristics, all 31 cohorts were maintained and evaluated as separate populations in all downstream genomic analyses. Unified bioinformatics processing and PCA confirmed the absence of confounding batch effects between sequencing datasets.

**Table 2:** Classification of the 31 chicken populations based on temperature adaptability and abdominal fat deposition traits.

Trait	Group	Included Chicken Populations
Temperature	Heat Tolerant Group	WCC, HBC, PC, XJC, GSBC, GSHC, SC, WDC, HTC, HYC, LYC, WHC, WHCA, WHCB, BYC, DWSC, YYDC, CJF, RJF
Temperature	Cold Tolerant Group	LDC, TLFGC, JBC, XCBBC
Temperature	Transitional Group	WL, RIRC, Cobb, WPR, WRR, SWFC, OC
Abdominal Fat	High Abdominal Fat Group	WCC, HBC, XJC, LYC, SC, HYC, WHC, WHCA, WHCB, XCBBC, PC
Abdominal Fat	Low Abdominal Fat Group	Cobb, WL, WPR, WRR, RIRC, SWFC, TLFGC, JBC, HTC, DWSC, GSBC, GSHC, WDC, LDC, BYC, OC
Abdominal Fat	Transitional Group	CJF, RJF

Classification of the 31 chicken populations based on temperature adaptability and abdominal fat deposition traits. The ‘Temperature’ trait categorizes populations into heat-tolerant, cold-tolerant, and transitional groups based on their historical geographic origin relative to the macro-ecological Qinling–Huaihe River boundary. The ‘Abdominal Fat’ trait stratifies populations into high, low, and transitional groups based on breed-specific slaughter performance data documented in Animal Genetic Resources in China: Poultry. For the full breed names corresponding to the population abbreviations, please refer to Table 1.

**Table 3:** Statistical analysis of the genotypic effects of the *MED17* downstream 166 bp DEL variant on abdominal fat traits.

SV Locus	Trait	0	1	2	*p*-Value	Effect Size ${(\eta }^{2})$
*MED17* downstream 166 bp DEL	Abdominal Fat Weight (g)	53.16 ± 5.79 ^a^ (*n* = 24)	67.26 ± 3.63 ^ab^ (*n* = 45)	72.15 ± 2.06 ^b^ (*n* = 138)	0.008	0.047
	Abdominal Fat Percentage (%)	2.79 ± 0.30 ^a^ (*n* = 24)	3.60 ± 0.19 ^ab^ (*n* = 45)	3.85 ± 0.11 ^b^ (*n* = 138)	0.005	0.051

Genotypes are coded as 0 (wild-type), 1 (heterozygous), and 2 (homozygous mutant). Data are presented as mean ± SE (adjusted for body weight). Different lowercase letters indicate significant differences between genotypes (*p* < 0.05).

## Data Availability

The whole-genome sequencing data analyzed in this study comprise both previously published datasets and newly generated sequences. The previously published data are publicly available, with detailed accession numbers and source references provided in Supplementary Table S1. The newly generated sequencing data are not yet deposited in an official public repository but are available to reviewers upon reasonable request to the corresponding author during the peer-review process. These newly generated data will be formally deposited in the NCBI Sequence Read Archive (https://www.ncbi.nlm.nih.gov/sra, accessed on 31 July 2026) and made publicly accessible upon manuscript acceptance.

## Supplementary Materials

The following supporting information can be downloaded at: https://www.mdpi.com/article/10.3390/ani16152417/s1.
