# Supplementary material for: Revealing the Shared Genetic Basis of Thermal Adaptation and Abdominal Adiposity in Wenchang Chicken Using Whole-Genome Structural Variation Analysis
Source: Animals (Basel). 2026 Aug 5;16(15):2417. doi: 10.3390/ani16152417 (PMC13465640; doi:10.3390/ani16152417)
Supplement: Supplementary file 1 [file animals-16-02417-s001.zip › Supplementary material.pdf]

Supplementary Materials

This study includes supplementary figures (S1–S6) and tables (S1–S5).

Supplementary Figures: Visualize population genetic structure (S1–S2), GO enrichment analysis (S3), *MED17* genotyping electrophoresis (S4), *MED17* tissue expression profiles (S5), and KEGG pathway signaling (S6).

Supplementary Tables: Provide sample sequencing information (S1), SV filtering statistics (S2), candidate genes for TA and AFD (S3), list of overlapping genes (S4), and functional enrichment results (S5).

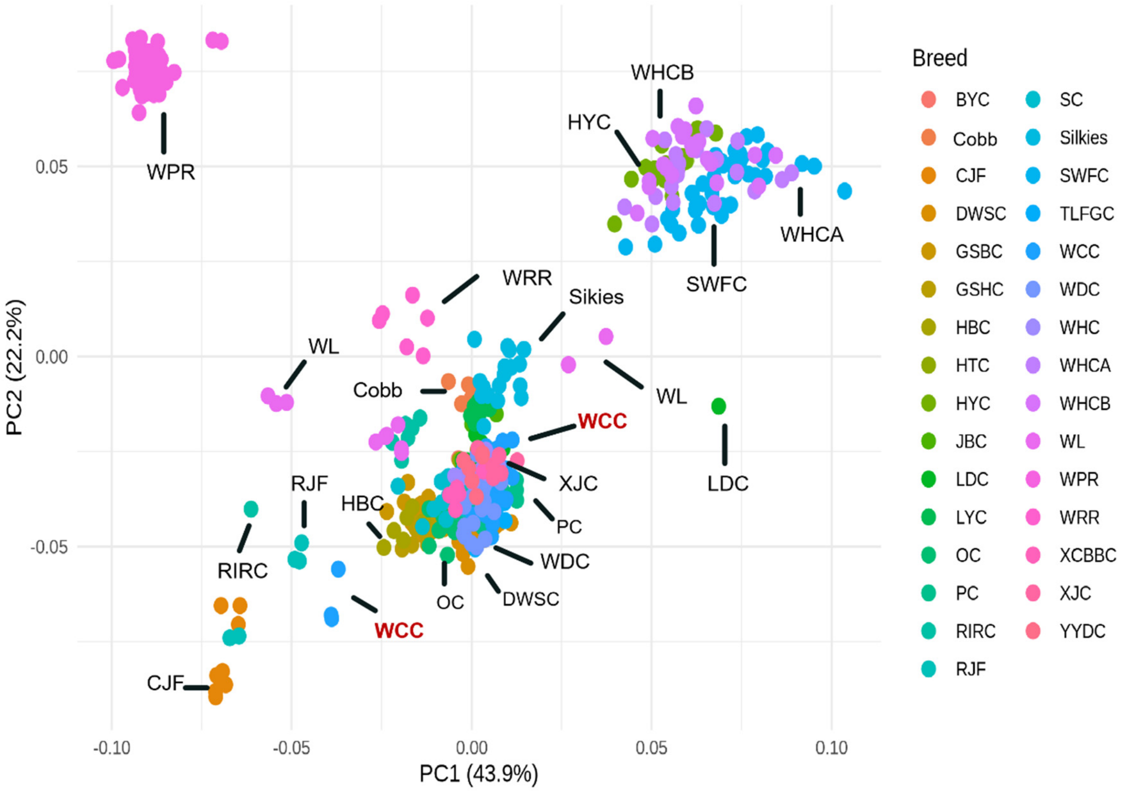

Figure S1. Principal component analysis (PCA) of 469 individuals across 31 global chicken populations. This plot illustrates the comprehensive genetic structure and relationships of all sampled populations, providing a broad context for the focused PCA analysis presented in Figure 1b. The first two principal components (PC1 and PC2) account for 43.9% and 22.2% of the genetic variance, respectively.

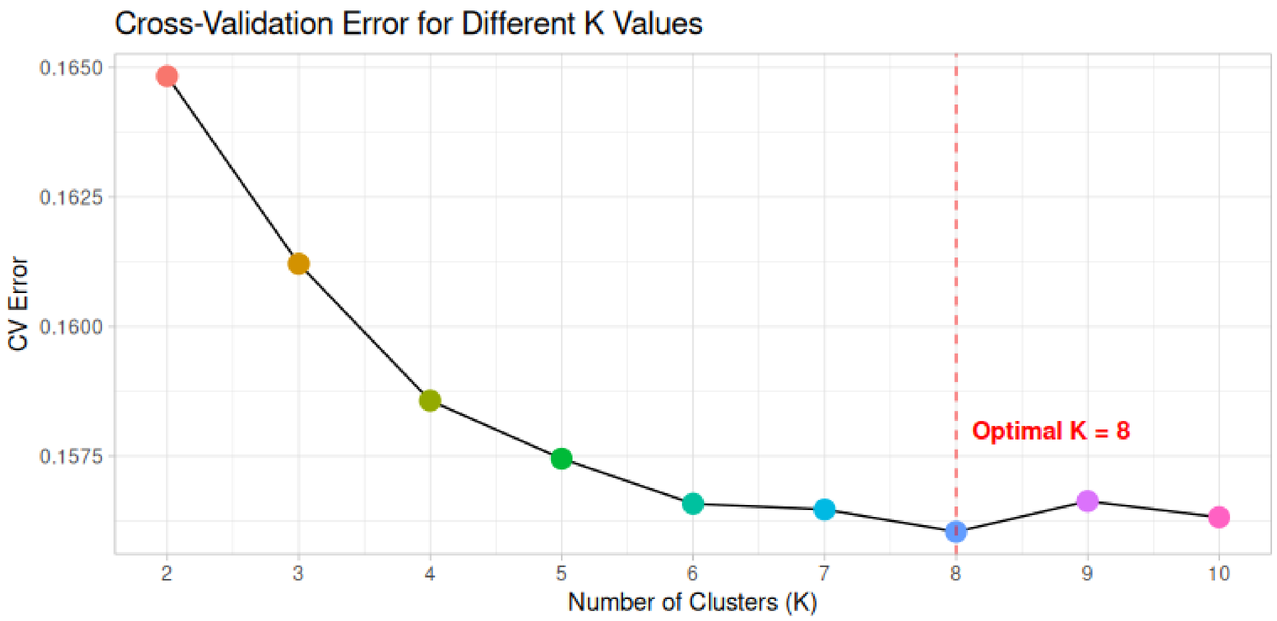

Figure S2 Cross-validation (CV) error estimation for determining the optimal number of genetic clusters (K). The plot displays the CV error values across different predefined K values (ranging from 2 to 10). The minimum CV error is observed at K = 8, indicating that the most probable number of ancestral populations for the analyzed dataset is 8.

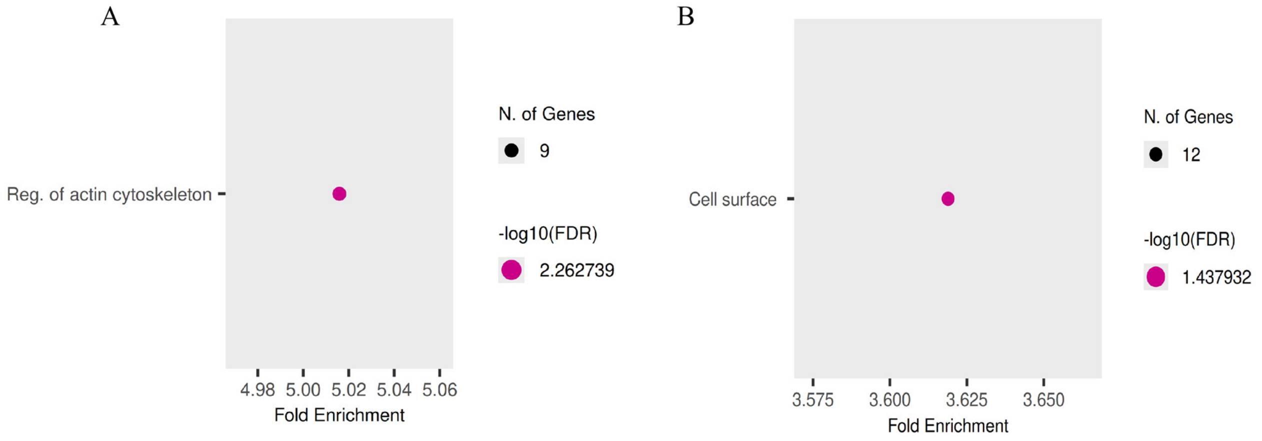

Figure S3. Gene Ontology (GO) enrichment analysis of the positively selected genes in the temperature-adapted group. The dot plots illustrate key enriched GO terms that support the physiological adaptation to thermal stress. (A) Significant enrichment in the biological process of

"Regulation of actin cytoskeleton". (B) Significant enrichment in the cellular component of "Cell surface". The x-axis represents the fold enrichment. The size of each dot corresponds to the number of genes mapped to the respective GO term, and the color intensity indicates the statistical significance defined by -log10(FDR).

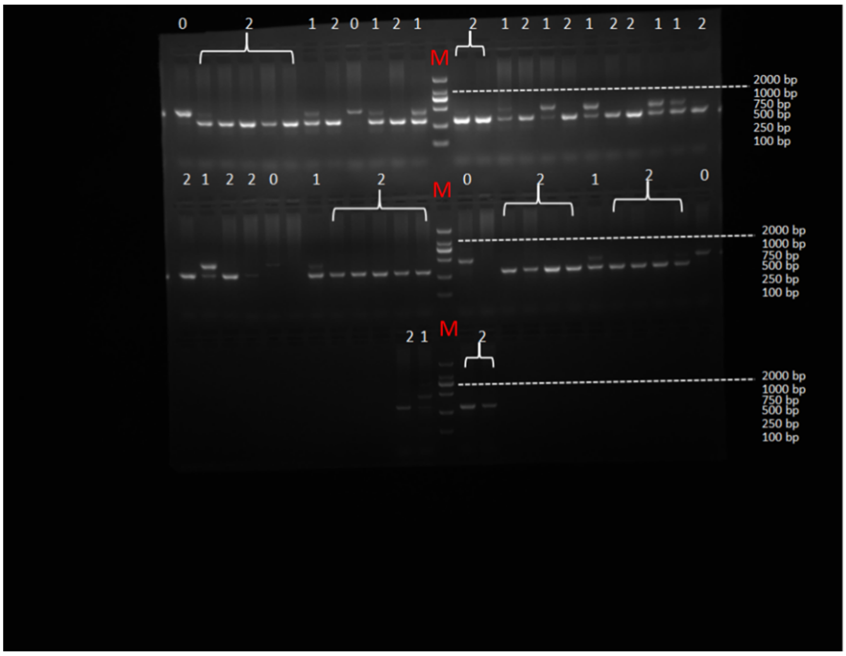

Figure S4 Original gel electrophoresis images of the MED17 SV genotyping. Representative gel images showing the PCR amplification products for the three genotypes: wild-type (0), heterozygous (1), and homozygous mutant (2). M indicates the DNA marker.

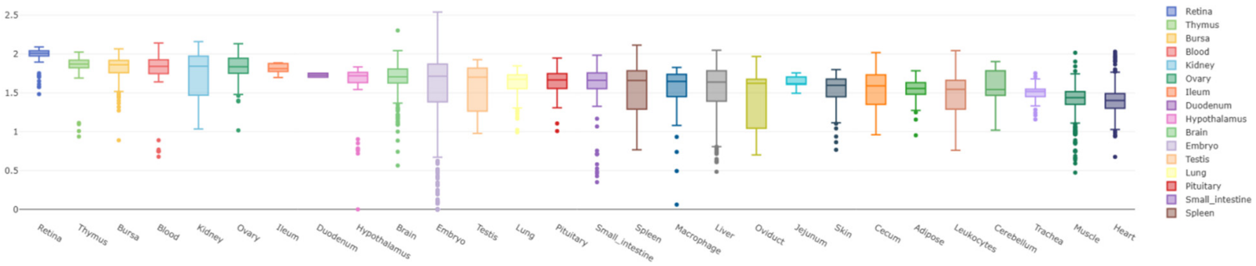

Figure S5. The multi-tissue gene expression profile of *MED17* in chickens. Data were retrieved from the ChickenGTEx Atlas database. The robust expression of *MED17* in central nervous tissues (e.g., Brain, Hypothalamus) and Adipose tissue provides transcriptional evidence supporting its pleiotropic regulatory role in both locomotor behavior and abdominal fat deposition.

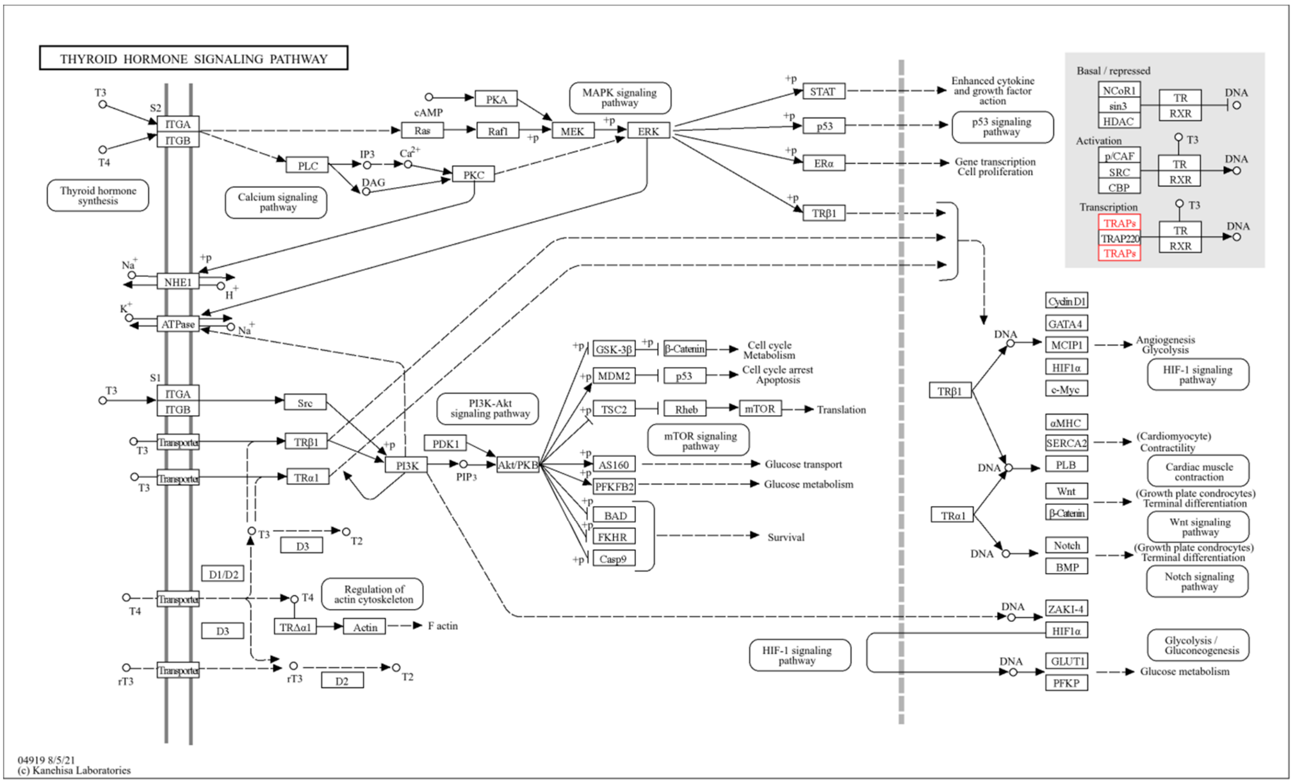

Figure S6. Complete KEGG pathway map for thyroid hormone signaling in Gallus gallus (gga04919). The position of the MED17 gene, is located within the TRAPs complex (highlighted in the red box on the upper right) within the nucleus. This complete map shows that MED17 acts as a crucial coactivator for the TR (Thyroid Hormone Receptor), bridging the hormone-receptor binding to downstream transcription of target genes, including those involved in Glucose metabolism and Thermogenesis. This complex regulates gene expression that is essential for energy homeostasis and adaptation to heat stress.

Additional file: Supplementary Tables S1–S5

Format: .xlsx

Description: This single Excel workbook contains four sheets providing detailed datasets supporting the study:

Table S1: Sample information and public sequencing data sources used in this study. This file details the genomic sequencing data retrieved from public databases. It includes the sample ID, breed abbreviation, NCBI BioProject ID, SRA accession number, and the original reference (DOI) for each individual sample analyzed in this study.

Table S2: Summary of structural variant (SV) filtering statistics. Description: This table summarizes the number of structural variants (SVs) before and after quality control filtering. It details the counts for five specific SV types—inversion, deletion, duplication, insertion, and breakend—and presents the total number of variants for both the initial (raw) and filtered datasets.

Table S3: Significant structural variations (SVs) and candidate genes identified by selection signature analysis in the temperature-adapted (TA) and abdominal fat deposition (AFD) groups.
Description: This file contains two sheets detailing the genome-wide selection signatures. The TA_Selection sheet lists the significantly selected SV loci and their nearest annotated genes associated with temperature adaptation. The AFD_Selection sheet details the significant SVs and candidate genes associated with abdominal fat deposition.

Table S4. Overlapping genes.the Overlapping_genes sheet provides the complete list of the 103 shared candidate genes identified across both the TA and AFD groups. This sheet provides comprehensive metrics including genomic coordinates (CHROM, POS), fixation index FST, log2-transformed nucleotide diversity ratio (log2ratio), and the nearest gene symbols.

Table S5. Functional enrichment analysis of candidate genes in the temperature-adapted (TA) and abdominal fat deposition (AFD) groups. This file contains multiple sheets detailing the Gene Ontology (GO) and KEGG pathway enrichment results. The sheets include: KEGG pathways for the TA group (TA_KEGG), GO Cellular Component for the TA group (TA_GO_CC), GO Molecular Function for the TA group (TA_GO_MF), and GO Biological Process for the AFD group (AFD_GO_BP). Abbreviations: TA, temperature-adapted; AFD, abdominal fat deposition.
